# Hepatitis B virus infection and development of chronic kidney disease: a cohort study

**DOI:** 10.1186/s12882-018-1154-4

**Published:** 2018-12-11

**Authors:** Yun Soo Hong, Seungho Ryu, Yoosoo Chang, Miguel Caínzos-Achirica, Min-Jung Kwon, Di Zhao, Tariq Shafi, Mariana Lazo, Roberto Pastor-Barriuso, Hocheol Shin, Juhee Cho, Eliseo Guallar

**Affiliations:** 1 Departments of Epidemiology and Medicine, and Welch Center for Prevention, Epidemiology, and Clinical Research, Johns Hopkins University Bloomberg School of Public Health, Baltimore, MD USA; 2 Center for Cohort Studies, Total Healthcare Center, Kangbuk Samsung Hospital, Sungkyunkwan University School of Medicine, Seoul, Republic of Korea; 3 Department of Health Sciences and Technology, Samsung Advanced Institute for Health, Sciences and Technology, Sungkyunkwan University, Seoul, Republic of Korea; 4 Department of Occupational and Environmental Medicine, Kangbuk Samsung Hospital, Sungkyunkwan University School of Medicine, Seoul, Republic of Korea; 5 Ciccarone Center for the Prevention of Heart Disease, Department of Cardiology, Johns Hopkins Medical Institutions, Baltimore, MD USA; 6 Bellvitge University Hospital, Barcelona, Spain; 7 RTI Health Solutions, Pharmacoepidemiology and Risk Management, Barcelona, Spain; 8 Department of Laboratory Medicine, Kangbuk Samsung Hospital, Sungkyunkwan University, School of Medicine, Seoul, South Korea; 9 Division of Nephrology, Department of Medicine, Johns Hopkins University School of Medicine, Baltimore, MD USA; 10 National Center for Epidemiology, Carlos III Institute of Health and Consortium for Biomedical Research in Epidemiology and Public Health (CIBERESP), Madrid, Spain; 11 Department of Family Medicine, Kangbuk Samsung Hospital and Sungkyunkwan University School of Medicine, Seoul, Republic of Korea

**Keywords:** Chronic kidney disease, Cohort study, Hepatitis B virus infection, Hepatitis B surface antigen, Proteinuria, Risk factors

## Abstract

**Background:**

The effect of chronic hepatitis B virus (HBV) infection on the risk of chronic kidney disease (CKD) is controversial. We examined the prospective association between hepatitis B surface antigen (HBsAg) serology status and incident CKD in a large cohort of men and women.

**Methods:**

Cohort study of 299,913 adults free of CKD at baseline who underwent health screening exams between January 2002 and December 2016 in South Korea. Incident CKD was defined as the development of an estimated glomerular filtration rate (eGFR) < 60 ml/min/1.73m^2^ and/or proteinuria.

**Results:**

Over 1,673,701 person-years of follow-up, we observed 13,924 incident cases of CKD (3225 cases of eGFR < 60 ml/min/1.73m^2^ and 11,072 cases of proteinuria). In fully adjusted models comparing positive to negative HBsAg participants, the hazard ratio (HR, 95% confidence interval) for incident CKD was 1.11 (1.03–1.21; *P* = 0.01). The corresponding HR for incident proteinuria and for eGFR < 60 ml/min/1.73m^2^ were 1.23 (1.12–1.35; *P* <  0.001) and 0.89 (0.73–1.07; *P* = 0.21), respectively. The associations were similar across categories of liver enzyme levels at baseline.

**Conclusion:**

In this large cohort, HBsAg positive serology was associated with higher risk of incident CKD, and we provide novel evidence that this association was due to a higher incidence of proteinuria in HBsAg positive participants. Our study adds to the growing body of evidence suggesting that chronic HBV infection may be a contributor to the increasing incidence of CKD.

**Electronic supplementary material:**

The online version of this article (10.1186/s12882-018-1154-4) contains supplementary material, which is available to authorized users.

## Background

Chronic hepatitis B virus (HBV) infection is one of the major causes of liver cirrhosis and hepatocellular carcinoma worldwide [1]. In addition to liver disease, HBV infection has been associated with extra-hepatic complications [2]. For example, various forms of kidney injury have been described in relation to HBV, including membranous nephropathy, membranoproliferative glomerulonephritis, and polyarteritis nodosa [3]. HBV-associated nephropathy most commonly presents with proteinuria or nephrotic syndrome [4], which may be caused by immune complex deposition, by virus-induced immunologic responses [3], or by direct glomerular and tubular injury by HBV [5]. It is unclear, however, whether exposure to HBV is associated with an increased risk of chronic kidney disease (CKD).

Previous longitudinal studies from Taiwan, where HBV infection is endemic, and China showed that positive hepatitis B surface antigen (HBsAg) serology was associated with increased risk of incident CKD [6, 7] and end-stage renal disease (ESRD) [8]. However, incident CKD and ESRD were identified from claims data using International Classification of Diseases, Ninth or Tenth Revision (ICD-9 or ICD-10), rather than using biomarkers of kidney function or kidney damage. The presence of hepatitis B core (anti-HBc) antibodies, on the contrary, was not associated with a higher incidence of CKD over 5 years of follow-up in a Chinese population undergoing screening exams [9]. The results from cross-sectional studies were also inconsistent, with most studies showing no association between HBV infection and the prevalence of CKD and one study showing an inverse association [10–13].

Chronic liver disease patients with coexisting renal impairment tend to have poorer outcomes than those with preserved renal function [14]. Therefore, early detection of CKD is of particular importance in the long-term management and prognosis of patients with chronic liver disease. We thus aimed to evaluate the prospective association between HBV infection and incident CKD, defined using estimated glomerular filtration rate (eGFR) and proteinuria, in a large cohort of men and women with normal renal function at baseline who participated in regular health screening examinations in South Korea.

## Methods

### Study population

The Kangbuk Samsung Health Study is a cohort study of adult men and women who underwent annual or biennial comprehensive medical health examinations at the two Kangbuk Samsung Hospital Total Healthcare Centers located in Seoul and Suwon, South Korea.

[15, 16].

**Fig. 1:**
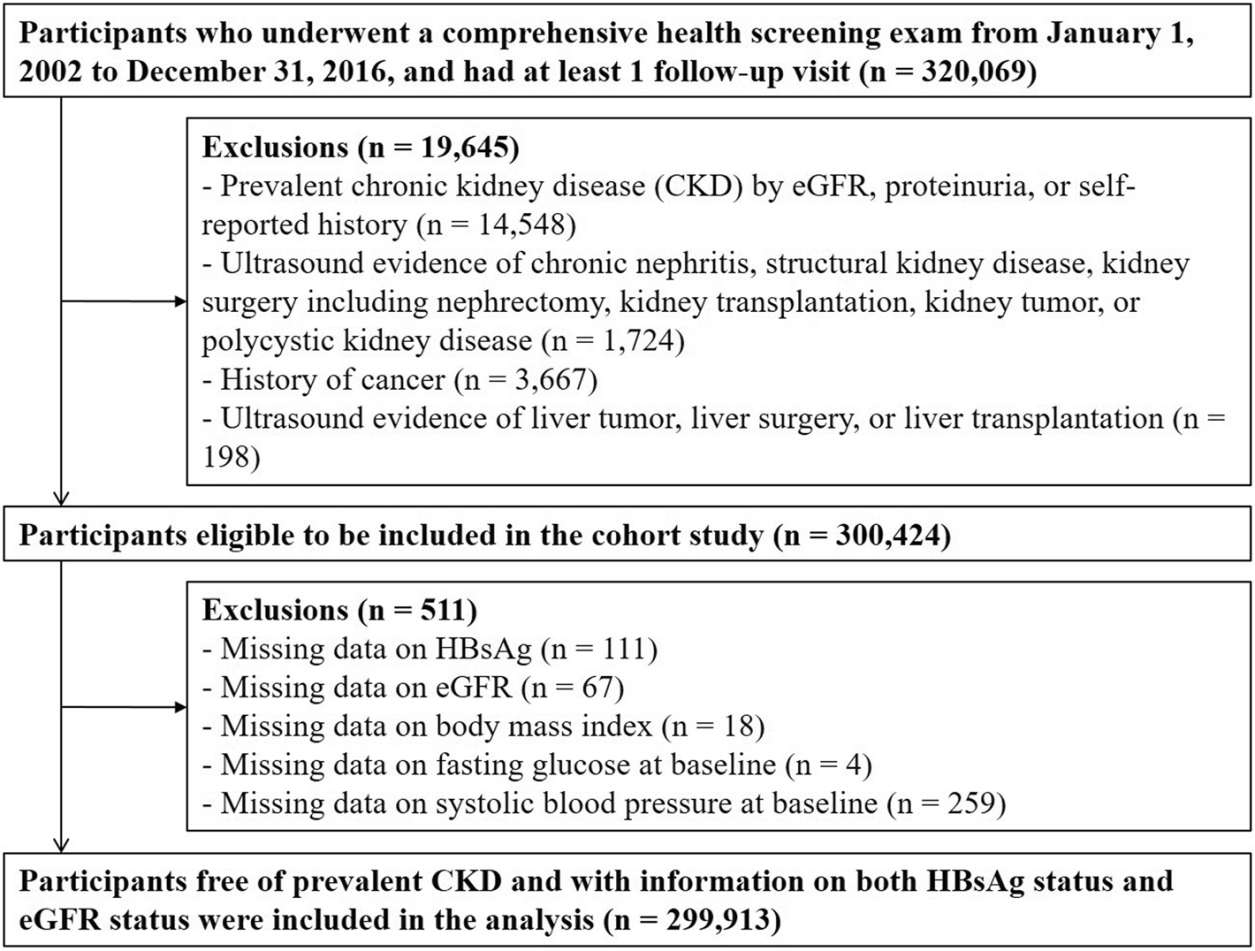
Flowchart of study participants Abbreviations: CKD, chronic kidney disease; eGFR, estimated glomerular filtration rate; HBsAg, Hepatitis B surface antigen

Among participants with at least one follow-up visit between January 1, 2002 and December 31, 2016 (*n* = 320,069), we excluded participants with any of the following conditions at baseline: prevalent CKD (*n* = 14,548), ultrasound evidence of chronic nephritis, structural kidney disease, kidney surgery, or kidney tumor, or kidney transplantation (*n* = 1724); self-reported history of cancer (*n* = 3667); ultrasound findings of liver tumor, liver surgery, or liver transplantation (*n* = 198). After excluding 19,645 participants, the number of eligible participants was 300,424 (170,214 men and 130,210 women). We further excluded participants with missing information on HBsAg serology, eGFR, body mass index (BMI), fasting blood glucose, or systolic blood pressure at baseline (*n* = 301). The final sample included 299,913 participants (169,994 men and 129,919 women; Fig. 1).

This study was approved by the Kangbuk Samsung Hospital Institutional Review Board that waived the requirement for informed consent as we only used de-identified data obtained as part of routine health screening exams.

### Data collection

As part of the comprehensive health exam, study participants provided detailed information on medical history, family history, medication use, smoking habits, alcohol intake, physical activity, and socioeconomic status at each visit using a standardized self-administered questionnaire at each visit. Smoking categories were defined as never, former, and current smokers. Current alcohol intake was estimated in grams per day. Physical activity was categorized based on the frequency of moderate- or vigorous-intensity exercise per week (< 3 or ≥ 3 times per week). Education level was categorized as less than college degree and college degree or higher.

Anthropometric measurements, including height, weight, and blood pressure, were measured at each visit by trained staff members under standard conditions. BMI was calculated as weight in kilograms divided by height in meters squared (kg/m^2^). Hypertension was defined as systolic blood pressure ≥ 140 mmHg, diastolic blood pressure ≥ 90 mmHg, self-reported history of hypertension, or self-reported use of antihypertensive medication.

The presence of fatty liver disease was determined at each visit by abdominal ultrasonography, which was a routine part of the health exam for all participants. Experienced radiologists at each center performed the exam using LOGIQ 700 MR machines with 3.5-MHz transducers (GE, Milwaukee, WI, USA). Fatty liver disease was diagnosed if there was diffuse hyperechoic parenchyma compared to that of the kidney or spleen [17]. Cirrhosis diagnosis was based on the presence of coarse and inhomogeneous parenchyma, caudate hypertrophy, surface nodularity, signs of portal hypertension, or regenerative nodules on ultrasonography.

### Laboratory determinations

At each visit, serum samples from all participants were tested for complete blood count, blood chemistry (including, but not limited to, renal function tests and liver function tests), and viral hepatitis serology, and urine samples were tested for presence of proteinuria.

Serum samples were analyzed for HBsAg using immunoradiometric assays (Radim, Via del Mare, Italy) in the Seoul center from 2002 to 2009 and in the Suwon center from 2002 to 2006, and using an electrochemiluminescent immunoassay (Modular E170; Roche Diagnostics) in both centers afterwards. A blood chemistry panel including alanine aminotransferase (ALT), aspartate aminotransferase (AST), gamma-glutamyl transferase (GGT), serum creatinine and serum glucose, was measured in fasting samples using Bayer Reagent Packs on an Advia 1650TM Autoanalyzer (Bayer Diagnostics, Medfeld, MA, USA) between 2002 and February 2010 at the Seoul center and between 2002 and September 2006 at the Suwon center, and on a Modular Analytics D2400 analyzer (Roche Diagnostics, Tokyo, Japan) in both centers afterwards.

Estimated GFR was calculated using the 4-variable Modification on Diet in Renal Disease Study equation, and CKD was defined as an eGFR < 60 ml/min/1.73m^2^ and/or the presence of proteinuria [18]. Diabetes was defined as a fasting serum glucose ≥126 mg/dl, a self-reported physician diagnosis, or current use of insulin or other hypoglycemic agents.

Urine dipsticks (URiSCAN Urine test strips, YD Diagnostics) were used to measure urine protein semi-quantitatively for all participants and were reported in 6 grades (absent, trace, 1+, 2+, 3+, and 4+). Presence of proteinuria was defined as grade 1+ or greater.

The Laboratory Medicine Department at Kangbuk Samsung Hospital has been accredited by the Korean Society of Laboratory Medicine (KSLM) and the Korean Association of Quality Assurance for Clinical Laboratories (KAQACL). The laboratory also participates in the survey proficiency testing provided by the College of American Pathologists (CAP).

### Statistical analysis

Baseline variables were summarized by HBsAg status as number (proportion) for categorical variables and mean (standard deviation) or median (interquartile range) for continuous variables, and compared using χ^2^ tests, Student’s t-tests, or signed-rank sum tests as appropriate.

Participants free of CKD at baseline were followed from the baseline visit until the development of CKD, or until the last screening visit. Development of CKD was evaluated at each visit based on the eGFR and the presence of proteinuria. Because the development of CKD was detected during a study visit but the exact date of its onset could not be determined, we used a parametric proportional hazards model to take into account this type of interval censoring (*stpm* command in Stata) [19]. The baseline hazard function was parametrized with restricted cubic splines in log time with four degrees of freedom. We estimated hazard ratios (HR) and 95% confidence intervals (CI) for incident CKD comparing HBsAg positive with HBsAg negative participants. To control for potential confounders, we used 3 models with progressive degrees of adjustment: Model 1 was adjusted for age, sex, study center, and baseline eGFR; Model 2 was further adjusted for smoking status, alcohol intake, level of education, physical activity, and BMI; and Model 3 was further adjusted for the presence of hypertension, diabetes, and fatty liver disease. We created indicator variables for missing values for smoking status (5.7%), alcohol intake (5.4%), level of education (26.5%), and physical activity (1.3%). There were no missing values for the presence of hypertension, diabetes, and fatty liver disease. The same analyses were also performed separately for the development of eGFR < 60 ml/min/1.73m^2^ and for the development of proteinuria.

We performed three sensitivity analyses. Elevated serum ALT levels indicate active inflammation of the liver cells and predict prognosis [20, 21]. Because the association of HBsAg status with kidney function may differ by the severity of inflammation in the liver, we stratified the analyses by baseline ALT level (elevated ALT defined as ALT > 41 U/l for males and ALT > 33 U/l for females). In addition, we excluded participants with evidence of liver cirrhosis on ultrasound because patients with liver cirrhosis are more susceptible to developing CKD [22–24]. Finally, we excluded participants who were positive for hepatitis C virus antibody (HCV Ab) because HCV infection can increase the risk of incident CKD [25, 26].

All statistical analyses were performed with Stata version 14.0 (StataCorp LP, College Station, Texas). Two-sided *P* values less than 0.05 were considered statistically significant.

## Results

The mean (SD) age of study participants was 37.3 (SD 7.9) years (Table 1). The prevalence of positive HBsAg serology was 3.7% (*n* = 11,209). Compared to seronegative individuals, those with positive HBsAg serology were more likely to be older, male, current smoker, and frequently engaged in vigorous exercise, to have higher levels of BMI, and liver enzymes, and to have lower alcohol intake, prevalence of fatty liver disease and eGFR levels. The prevalence of hypertension and diabetes were not significantly different between those with and without HBsAg (Table 1).

**Table 1:** Participant characteristics by hepatitis B virus infection at baseline (*n* = 299,913)

Characteristics	Overall	Hepatitis B virus infection		*P* value
		HBsAg (−)	HBsAg (+)	
Number (%)	299,913	288,704 (96.3)	11,209 (3.7)	
Age, years^*^	37.3 (7.9)	37.3 (7.9)	38.2 (7.6)	< 0.001
Men, %	56.7	56.4	63.7	< 0.001
Current smoker, %	23.6	23.6	25.1	< 0.001
Alcohol intake, g/day^†^	5 (0–15)	5 (0–15)	4 (0–13)	< 0.001
Vigorous exercise, %^‡^	14.2	14.2	15.7	< 0.001
Higher education, %^§^	58.5	58.5	58.1	0.57
BMI, kg/m^2*^	23.2 (3.2)	23.2 (3.2)	23.5 (3.1)	< 0.001
ALT, U/l^†^	19 (14–29)	19 (14–29)	26 (18–38)	< 0.001
AST, U/l^†^	21 (18–26)	21 (17–26)	25 (20–31)	< 0.001
GGT, U/l^†^	19 (12–33)	19 (12–33)	20 (13–33)	< 0.001
eGFR, ml/min/1.73m^2*^	88.2 (16.6)	88.3 (16.7)	86.4 (15.2)	< 0.001
Glucose, mg/dl^*^	93.6 (13.7)	93.6 (13.7)	93.1 (13.6)	< 0.001
Hypertension, %	11.7	11.7	12.0	0.37
Diabetes, %	2.4	2.4	2.5	0.38
Fatty liver disease, %	25.4	25.6	22.7	< 0.001

Values are *mean (standard deviation), ^†^median (interquartile range), or percentage

^‡^Moderate- or vigorous-intensity exercise ≥3 times per week

^§^College graduate or higher

Abbreviations: *ALT* alanine aminotransferase, *AST* aspartate aminotransferase, *BMI* body mass index, *eGFR* estimated glomerular filtration rate, *GGT* gamma-glutamyl transferase

The mean duration of follow-up was 5.6 years (1,673,701 person-years of follow-up). Overall, there were 13,924 new cases of CKD (incidence rate 8.3 per 1000 person-years). By different definitions of CKD, there were 3225 new cases of eGFR < 60 ml/min/1.73m^2^ and 11,072 new cases of proteinuria during follow-up (incidence rates of 1.9 and 6.6 per 1000 person-years, respectively). Participants who developed CKD were older, had higher levels of liver enzymes and serum glucose, had lower eGFR levels, and were more likely to have other comorbidities, such as hypertension, diabetes, and fatty liver disease at baseline (Additional file 1: Table S1).

**Fig. 2:**
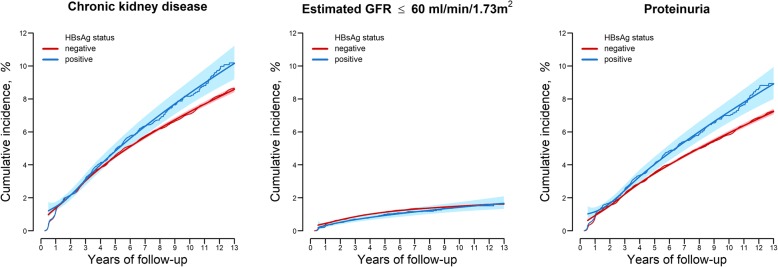
Adjusted cumulative incidence of chronic kidney disease by HBsAg serology at baseline Parametric cumulative incidence curves (smooth lines) were estimated from a spline-based parametric survival model allowing for non-proportional hazards between positive HBsAg and negative HBsAg groups. Nonparametric cumulative incidence curves (step functions) were estimated from Kaplan-Meier methods. Both methods were weighted by stabilized inverse probability weights and stratified by HBsAg serology status. Confounders used to estimate inverse probability weights were measured at baseline, and included age (< 30, 30–34, 35–39, 40–44, 45–49, 50–54, 55–59 or ≥ 60 years), sex (male or female), study center (Seoul or Suwon), year of health screening exam, eGFR (< 90 or ≥ 90 mL/min/1.73m^2^), smoking status (never, former, or current), alcohol intake (none, moderate, or high), education (less than college degree, or college degree or higher), exercise (< 3 or ≥ 3 times per week of moderate- or vigorous-intensity exercise), BMI (< 18.5, 18.5–22.9, 23–24.9, or ≥ 25 kg/m^2^), presence of diabetes, presence of hypertension, and presence of fatty liver disease. Abbreviations: BMI, body mass index; CKD, chronic kidney disease; eGFR, estimated glomerular filtration rate; HBsAg, Hepatitis B surface antigen

There were 609 incident cases of CKD among 11,209 HBsAg positive participants and 13,315 incident cases of CKD among 288,704 HBsAg negative participants (incidence rates of 9.3 and 8.3 per 1000 person-years, respectively; Table 2). In fully adjusted models, the HR comparing HBsAg positive participants to HBsAg negative participants was 1.11 (95% CI 1.03–1.21; Table 2 and Fig. 2). In additional analyses excluding 215 participants who reported ever having been treated for viral hepatitis at baseline or over follow-up, the fully adjusted HR for incident CKD comparing HBsAg positive participants to HBsAg negative participants was 1.11 (95% CI 1.02–1.21).

**Table 2:** Hazard ratios (HR) for incident chronic kidney disease by HBsAg serology (*n* = 299,913)

	No. of incident cases (person-years)	Model 1 HR (95% CI)	Model 2 HR (95% CI)	Model 3 HR (95% CI)
eGFR < 60 ml/min/1.73m^2^ or proteinuria				
HBsAg (−)	13,315 (1,608,299.2)	1.00 (reference)	1.00 (reference)	1.00 (reference)
HBsAg (+)	609 (65,401.8)	1.07 (0.99–1.16)	1.09 (1.00–1.18)	1.11 (1.03–1.21)
eGFR < 60 ml/min/1.73m^2^				
HBsAg (−)	3106 (1,641,700.4)	1.00 (reference)	1.00 (reference)	1.00 (reference)
HBsAg (+)	119 (67,044.5)	0.91 (0.76–1.09)	0.87 (0.72–1.05)	0.89 (0.73–1.07)
**Proteinuria**				
HBsAg (−)	10,560 (1,621,635.3)	1.00 (reference)	1.00 (reference)	1.00 (reference)
HBsAg (+)	512 (65,806.8)	1.17 (1.07–1.28)	1.20 (1.10–1.31)	1.23 (1.12–1.35)

Model 1: adjusted for age, sex, center, and baseline eGFR; Model 2: further adjusted for smoking (never, former and current), alcohol intake (g/day), level of education (high school graduate or less and college graduate or higher), physical activity (moderate- or vigorous-intensity physical activity < 3 times/week and ≥ 3 times/week), and BMI (kg/m^2^); and Model 3: further adjusted for hypertension, diabetes, and presence of fatty liver disease

When the analyses were performed separately for each CKD component, the association between HBsAg serology and kidney outcomes was limited to incident proteinuria. In fully adjusted models, the HR for incident proteinuria comparing HBsAg positive to HBsAg negative participants was 1.23 (95% CI 1.12–1.35), whereas the corresponding HR for eGFR < 60 ml/min/1.73m^2^ was 0.89 (95% CI 0.73–1.07; Table 2 and Fig. 2). After excluding participants who reported ever having been treated for viral hepatitis at baseline or over follow-up, the corresponding HR for incident proteinuria was 1.23 (95% CI 1.12–1.35;) and for eGFR < 60 ml/min/1.73m^2^ was 0.88 (95% CI 0.73–1.07).

In sensitivity analyses, the association between HBsAg serology and kidney outcomes was similar in participants with normal and with elevated ALT levels at baseline (Additional file 1: Table S2). Similarly, the findings were essentially unchanged after excluding participants with ultrasound evidence of liver cirrhosis (Additional file 1: Table S3) and after excluding participants with positive HCV Ab serology (Additional file 1: Table S4).

## Discussion

In this large cohort of adults without clinically apparent kidney disease, HBsAg positive participants had a higher incidence risk of CKD compared to HBsAg negative participants. The increased risk was driven by development of proteinuria, although the number of HBsAg positive participants who developed eGFR < 60 ml/min/1.73 m^2^ was small. The results were similar in participants with normal or with elevated liver enzyme levels at baseline, and after excluding participants with cirrhosis or with positive HCV antibodies. Since chronic hepatitis B infection is highly prevalent in many countries, our findings suggest that it may be contributing to the global burden of CKD.

The role of hepatitis B infection in the development of CKD is controversial. In a meta-analysis of 4 cohort studies, the pooled HR for CKD comparing participants with HBV infection to those without HBV infection was 2.2 (95% CI 0.95–3.50) [27], but there was substantial heterogeneity across studies. In another meta-analysis of cohort, cross-sectional, and case-control studies in Asian populations, there was no association between HBsAg serology and reduced eGFR (adjusted risk ratio 0.95; 95% CI 0.72–1.26) or proteinuria (adjusted risk ratio 1.00; 95% CI 0.83–1.20) [28].

The largest study in both meta-analyses, a nationwide cohort from Taiwan that used claims data to evaluate the association between chronic HBV infection with incident CKD [6] and incident ESRD [8], found very high HRs (2.58 and 3.85 for CKD and ESRD, respectively). In the large China Kadoori Biobank cohort, participants with HBsAg had higher incidence of CKD compared to participants without HBsAg (adjusted HR 1.37; 95% CI 1.18–1.60). CKD was defined using ICD-10 codes from national health insurance system claims data [7]. Claims-based studies may have limited sensitivity to detect asymptomatic HBV infection, and are likely to capture more severe, symptomatic cases of HBV infection and kidney diseases. Claims-based data have also a limited ability to identify participants with prevalent CKD at baseline and with asymptomatic or early stages of chronic kidney disease, and they are prone to surveillance bias as patients with a diagnosis of HBV infection may be more likely to be tested for kidney function, and vice versa. Furthermore, using ICD codes does not distinguish whether incident CKD was mainly caused by reduced eGFR or by the development of proteinuria. In our study, we used repeated measurements of eGFR and proteinuria in participants undergoing regular health screening exam to define incident CKD, which allowed us to evaluate the different mechanisms of developing CKD separately and to identify CKD in otherwise healthy participants.

In a smaller cohort of health screening examinees in China, there was no association between the presence of anti-HBc antibodies and the incidence of reduced eGFR, proteinuria, or CKD over a 5-year period. The presence of anti-HBc antibodies, however, cannot differentiate resolved HBV infections from chronic active HBV infections [9]. In our analysis, we used a positive HBsAg test as a marker of exposure. In East Asian countries, including China and Korea, HBV is most often transmitted vertically at birth, and a positive HBsAg test in adults most likely represents chronic HBV infection [29].

In three cross-sectional studies conducted also in East Asian countries, positive HBsAg serology was not associated with an increased risk of prevalent proteinuria [11, 13, 30]. In contrast, the significant association between HBsAg serology and incidence of CKD in the present study was due to incident proteinuria, with no clear association between HBsAg serology and the incidence of reduced eGFR. Our prospective study suggests that the increased incidence of CKD in HBsAg positive subjects is mainly due to an increased incidence of proteinuria. This finding is also supported by the fact that HBV-associated nephropathies, such as membranous nephropathy and membranoproliferative glomerulonephritis, most commonly present with proteinuria or nephrotic syndrome.

The prevalence of CKD among HBsAg positive subjects ranges from 0.4 to 11.4%, but it tends to be higher among those with elevated ALT [10, 11, 31]. In our study, the incidence of CKD was higher in participants with elevated ALT levels (12.2 per 1000 person-years) than in participants with normal ALT levels at baseline (7.7 per 1000 person-years). HBV-associated nephropathy is more common when there is active replication of the virus or active inflammation in liver cells (immune tolerant or immune clearance phase) than when the viral burden and liver enzyme levels are low (inactive carrier phase) [3]. In our study, however, the association between positive HBV serology and incidence of CKD was similar across baseline ALT level categories, suggesting that the risk of kidney damage is elevated in all stages of HBV infection, even in the absence of active viral replication or inflammation.

Our findings are consistent with those from basic research studies. HBV-associated nephropathy is mainly due to immunological processes, particularly immune complex deposition in the kidney [3]. The circulating antigen-antibody complex formed at the acute exposure to HBV may continue to damage the glomerular structure even when the virus is not actively replicating, leading to proteinuria. Indeed, the most common types of HBV-associated nephropathy, such as membranous nephropathy and membranoproliferative glomerulonephritis, involve proteinuria. In addition to immunologic mechanisms, the virus may damage the kidney either directly or through apoptosis. HBV DNA has been identified both in glomerular and in tubular cells [3, 5], and it may promote apoptosis of renal tubular cells through upregulation of the Fas pathway [32]. In our study, we did not have histologic diagnosis of the type of kidney damage or information about potential mechanistic pathways. Additional studies are needed to better understand the mechanisms underlying the association between HBV infection and kidney damage.

There are a few limitations to our study. We used urine dipsticks to identify proteinuria, but dipsticks may not be sensitive enough to detect low levels of proteinuria [33]. Furthermore, we defined CKD using a single measurement of eGFR and/or proteinuria, whereas the current guideline defines CKD as abnormalities or markers of damage for at least 3 months [34]. These sources of measurement error, however, are random with respect to exposure and would tend to underestimate the underlying associations. Second, our study may not have been long enough to see the effect of HBV on reduction in eGFR, especially since our participants were relatively young and healthy with stable liver function and low prevalence of underlying comorbidities. Third, we did not have information on the presence of hepatitis B e-antigen, on HBV DNA titers, or detailed history on HBV treatment (although we know which participants received treatment for viral hepatitis from self-reports). Treatment with oral antiviral agents may decrease renal function in chronic hepatitis B patients, whereas their effects on the development of proteinuria is less certain [35, 36] Tenofovir, which may cause both reduction in renal function and tubular damage, was introduced to Korea in 2012 and is unlikely to be the cause of higher incidence of proteinuria in HBsAg positive participants in our study. In addition, excluding participants who reported having ever been treated for viral hepatitis at baseline or over follow-up did not change our results. Finally, our study population was comprised of Korean men and women participating in regular health screening exams and our findings may not generalize to other race/ethnicity groups.

There are also several strengths to the study. In addition to the large sample size, our study participants were relatively young and healthy. As a result, the association between HBsAg and incident CKD is less likely to be confounded by comorbidities and medication use than studies conducted in elderly cohorts. In addition, detailed health screening information on anthropometric measures, lifestyle behaviors, medical history, and laboratory tests allowed us to account for multiple potential confounders. Finally, we measured urine protein in addition to eGFR and we were able to evaluate the association of HBsAg with eGFR and with proteinuria separately.

## Conclusion

In conclusion, our study provides evidence to support an association between HBsAg positive serology and higher incidence of CKD. More specifically, we provide novel evidence that this association is due to a higher incidence of proteinuria in HBsAg positive compared to HBsAg negative subjects. Studies with detailed information on HBV replication status, HBV treatment history, and longer follow-up are needed to provide further insight to the association between HBV and CKD. Because patients with both chronic liver disease and CKD have much poorer prognosis and higher mortality than those with either condition, prevention and early detection of kidney disease is essential in patients with chronic HBV infection.

## Additional files

Additional file 1: **Table S1.** Baseline participant characteristics by incidence of chronic kidney disease (*n* = 299,913).**Table S2.** Hazard ratios (HR) for incident chronic kidney disease by HBsAg serology by ALT status at baseline. **Table S3.** Hazard ratios (HR) for incident chronic kidney disease (eGFR < 60 ml/min/1.73m^2^ and/or proteinuria) by HBsAg serology among participants without liver cirrhosis at baseline (*n* = 299,264). **Table S4.** Hazard ratios (HR) for incident chronic kidney disease (eGFR < 60 ml/min/1.73m^2^ and/or proteinuria**)** by HBsAg serology among participants without HCV Ab at baseline (*n* = 294,377). (DOCX 35 kb)

## Abbreviations

anti-HBc antibody: Antibody to hepatitis B core antigen
BMI: Body Mass Index
CKD: Chronic Kidney Disease
eGFR: Estimated Glomerular Filtration Rate
ESRD: End-stage Renal Disease
HBsAg: Hepatitis B surface Antigen
HBV: Hepatitis B Virus
HCV: Hepatitis C Virus
