# Supplementary material for: Hepatitis B virus infection and development of chronic kidney disease: a cohort study
Source: BMC Nephrol. 2018 Dec 11;19:353. doi: 10.1186/s12882-018-1154-4 (PMC6288894; doi:10.1186/s12882-018-1154-4)
Supplement: Supplementary file 1 — Table S1. Baseline participant characteristics by incidence of chronic kidney disease (n = 299,913).Table S2. Hazard ratios (HR) for incident chronic kidney disease by HBsAg serology by ALT status at baseline. Table S3. Hazard ratios (HR) for incident chronic kidney disease (eGFR < 60 ml/min/1.73m2 and/or proteinuria) by HBsAg serology among participants without liver cirrhosis at baseline (n = 299,264). Table S4. Hazard ratios (HR) for incident chronic kidney disease (eGFR < 60 ml/min/1.73m2 and/or proteinuria) by HBsAg serology among participants without HCV Ab at baseline (n = 294,377). (DOCX 35 kb) [file 12882_2018_1154_MOESM1_ESM.docx]

**Supplementary Table 1.** Baseline participant characteristics by incidence of chronic kidney disease (*n* = 299,913).

| **Characteristics** | **Incident CKD** | | ***P* value** |
| --- | --- | --- | --- |
|  | **No** | **Yes** |  |
| Number | 285,989 | 13,924 |  |
| Age, years^†^ | 37.3 (7.9) | 38.9 (8.5) | < 0.001 |
| Men, % | 56.3 | 64.9 | < 0.001 |
| Current smoker, % | 23.3 | 31.1 | < 0.001 |
| Alcohol intake, g/day^‡^ | 5 (0 – 15) | 6 (0 – 15) | < 0.001 |
| Vigorous exercise, %^§^ | 14.1 | 16.1 | < 0.001 |
| Higher education, %^¶^ | 58.5 | 57.1 | < 0.001 |
| BMI, kg/m^2†^ | 23.2 (3.2) | 24.1 (3.5) | < 0.001 |
| ALT, U/l^‡^ | 19 (14 – 29) | 23 (16 – 35) | < 0.001 |
| AST, U/l^‡^ | 21 (17 – 26) | 23 (19 – 29) | < 0.001 |
| GGT, U/l^‡^ | 19 (12 – 33) | 24 (14 – 44) | < 0.001 |
| eGFR, mg/min/1.73m^2†^ | 88.6 (16.6) | 80.8 (14.7) | < 0.001 |
| Glucose, mg/dl^†^ | 93.5 (13.2) | 96.7 (21.4) | < 0.001 |
| Hypertension, % | 11.3 | 20.9 | < 0.001 |
| Diabetes, % | 2.2 | 6.0 | < 0.001 |
| Fatty liver disease, % | 25.1 | 34.4 | < 0.001 |
| Positive HBsAg, % | 3.7 | 4.4 | < 0.001 |

Values are ^†^mean (standard deviation), ^‡^median (interquartile range), or percentage.

^§^ Moderate- or vigorous-intensity exercise ≥ 3 times per week.

^¶^ College graduate or higher.

Abbreviations: ALT, alanine aminotransferase; AST, aspartate aminotransferase; BMI, body mass index; CKD, chronic kidney disease; eGFR, estimated glomerular filtration rate; GGT, gamma-glutamyl transferase.

**Supplementary Table 2.** Hazard ratios (HR) for incident chronic kidney disease by HBsAg serology by ALT status at baseline.

**Normal ALT at baseline (*n* = 262,575)**

|  | **Model 1**  **HR (95% CI)** | **Model 2**  **HR (95% CI)** | **Model 3**  **HR (95% CI)** |
| --- | --- | --- | --- |
| **eGFR < 60 ml/min/1.73m^2^** **or proteinuria** |  |  |  |
| HBsAg (-) | 1.00 (reference) | 1.00 (reference) | 1.00 (reference) |
| HBsAg (+) | 1.05 (0.95 – 1.16) | 1.06 (0.96 – 1.18) | 1.08 (0.98 – 1.20) |
| **eGFR < 60 ml/min/1.73m^2^** |  |  |  |
| HBsAg (-) | 1.00 (reference) | 1.00 (reference) | 1.00 (reference) |
| HBsAg (+) | 0.98 (0.85 – 1.12) | 0.95 (0.82 – 1.09) | 0.97 (0.84 – 1.12) |
| **Proteinuria** |  |  |  |
| HBsAg (-) | 1.00 (reference) | 1.00 (reference) | 1.00 (reference) |
| HBsAg (+) | 1.23 (1.16 – 1.31) | 1.25 (1.17 – 1.33) | 1.27 (1.19 – 1.35) |

Model 1: adjusted for age, sex, center, and baseline eGFR; Model 2: further adjusted for smoking (never, former and current), alcohol intake (g/day), level of education (high school graduate or less and college graduate or higher), physical activity (moderate- or vigorous-intensity physical activity < 3 times/week and ≥ 3 times/week), and BMI (kg/m^2^); and Model 3: further adjusted for hypertension, diabetes, and presence of fatty liver disease.

**Elevated ALT (*n* = 37,236)**

|  | **Model 1**  **HR (95% CI)** | **Model 2**  **HR (95% CI)** | **Model 3**  **HR (95% CI)** |
| --- | --- | --- | --- |
| **eGFR < 60 ml/min/1.73m^2^** **or proteinuria** |  |  |  |
| HBsAg (-) | 1.00 (reference) | 1.00 (reference) | 1.00 (reference) |
| HBsAg (+) | 0.99 (0.85 – 1.14) | 1.10 (0.94 – 1.28) | 1.15 (0.99 – 1.34) |
| **eGFR < 60 ml/min/1.73m^2^** |  |  |  |
| HBsAg (-) | 1.00 (reference) | 1.00 (reference) | 1.00 (reference) |
| HBsAg (+) | 1.03 (0.82 – 1.29) | 1.08 (0.86 – 1.36) | 1.10 (0.87 – 1.38) |
| **Proteinuria** |  |  |  |
| HBsAg (-) | 1.00 (reference) | 1.00 (reference) | 1.00 (reference) |
| HBsAg (+) | 1.04 (0.94 – 1.15) | 1.18 (1.07 – 1.31) | 1.24 (1.11 – 1.38) |

Model 1: adjusted for age, sex, center, and baseline eGFR; Model 2: further adjusted for smoking (never, former and current), alcohol intake (g/day), level of education (high school graduate or less and college graduate or higher), physical activity (moderate- or vigorous-intensity physical activity < 3 times/week and ≥ 3 times/week), and BMI (kg/m^2^); and Model 3: further adjusted for hypertension, diabetes, and presence of fatty liver disease.

**Supplementary Table 3.** Hazard ratios (HR) for incident chronic kidney disease (eGFR < 60 ml/min/1.73m^2^ and/or proteinuria) by HBsAg serology among participants without liver cirrhosis at baseline (*n* = 299,264).

|  | **Model 1**  **HR (95% CI)** | **Model 2**  **HR (95% CI)** | **Model 3**  **HR (95% CI)** |
| --- | --- | --- | --- |
| **Without liver cirrhosis** |  |  |  |
| HBsAg (-) | 1.00 (reference) | 1.00 (reference) | 1.00 (reference) |
| HBsAg (+) | 1.07 (0.99 – 1.16) | 1.09 (1.00 – 1.18) | 1.11 (1.02 – 1.21) |

Model 1: adjusted for age, sex, center, and baseline eGFR; Model 2: further adjusted for smoking (never, former and current), alcohol intake (g/day), level of education (high school graduate or less and college graduate or higher), physical activity (moderate- or vigorous-intensity physical activity < 3 times/week and ≥ 3 times/week), and BMI (kg/m^2^); and Model 3: further adjusted for hypertension, diabetes, and presence of fatty liver disease.

**Supplementary Table 4.** Hazard ratios (HR) for incident chronic kidney disease (eGFR < 60 ml/min/1.73m^2^ and/or proteinuria**)** by HBsAg serology among participants without HCV Ab at baseline (*n* = 294,377).

|  | **Model 1**  **HR (95% CI)** | **Model 2**  **HR (95% CI)** | **Model 3**  **HR (95% CI)** |
| --- | --- | --- | --- |
| **Without HCV Ab** |  |  |  |
| HBsAg (-) | 1.00 (reference) | 1.00 (reference) | 1.00 (reference) |
| HBsAg (+) | 1.08 (0.99 – 1.17) | 1.09 (1.01 – 1.19) | 1.12 (1.03 – 1.22) |

Model 1: adjusted for age, sex, center, and baseline eGFR; Model 2: further adjusted for smoking (never, former and current), alcohol intake (g/day), level of education (high school graduate or less and college graduate or higher), physical activity (moderate- or vigorous-intensity physical activity < 3 times/week and ≥ 3 times/week), and BMI (kg/m^2^); and Model 3: further adjusted for hypertension, diabetes, and presence of fatty liver disease.
